# The impact of geriatric syndromes on quality of life among older people living with HIV in Kampala, Uganda

**DOI:** 10.3389/fpubh.2024.1306151

**Published:** 2024-01-23

**Authors:** Elizabeth Senkoro, Phoebe Mbabazi, Grace Banturaki, Suzan Naikoba, Barbara Castelnuovo

**Affiliations:** ^1^Mark Wainberg Fellowship Program, International AIDS Society (IAS), Geneva, Switzerland; ^2^Infectious Diseases Institute, College of Health Sciences, Makerere University, Kampala, Uganda; ^3^Chronic Disease Clinic, Ifakara Health Institute, Ifakara, Tanzania

**Keywords:** older people with HIV, quality of life, sub-Saharan Africa, geriatric syndromes, WHOQOL – OLD

## Abstract

**Objective:**

Older people living with HIV (OPWH) often have lower quality of life (QoL) compared to general population. Measuring their QoL is an important step in HIV care to ensure they have long healthy lives. This study aimed to evaluate the quality of life and its associated factors among people living with HIV aged 60 years and above in Uganda.

**Methods:**

We used a cross-sectional analysis of older people living with HIV (OPWH) enrolled in a prospective cohort from December 2020 – December 2021. Quality of life was assessed using the World Health Organisation QoL OLD instrument (WHOQOL-OLD). Linear regression model was used to determine associated factors.

**Results:**

Of the 500 participants enrolled, 51.2% were men and their median age was 64 years (IQR: 62 — 68). WHOQOL-OLD mean score (SD) was 90.1 (8.3) out of 120. Factors that increased overall QoL were (Coefficient [95% Confidence Interval]): being male 2.35 (1.21 — 3.73), having an income of ≥$1 1.30 (−0.16 — 2.76) and paradoxically having more than 2 non-communicable diseases 0.69 (−0.76 — 2.14) in the past, present and future domain of QoL. Those that decreased QoL in the overall and various domains included: an increasing number of geriatric syndromes, depression, pre-frailty, frailty, malnutrition, and low physical function.

**Conclusion:**

Our findings suggest that financial stability contributed to good QoL while geriatric syndromes decreased QoL for OPWH. Integrating the screening and management of geriatric syndromes into HIV care has the potential to improve the overall QoL of OPWH.

## Introduction

People living with HIV (PLWH) are growing older and their life span has significantly increased over the past four decades as a result of highly active antiretroviral treatment. This has changed the deadly HIV infection into a chronic condition (1, 2). Although the proportion of older people living with HIV (OPWH) is greater in the global North than in sub-Saharan Africa (SSA), the number of OPWH in SSA is expected to triple by 2040 (1, 3). This growth is attributed to improved access to effective HIV care and treatment programs (2) and the increase in new HIV infections among older adults (4, 5). For instance, in Uganda, HIV prevalence in all age groups declined in 2017 compared to 2020 except for those ≥50 years (6, 7).

There has been a growing call for the expansion of the 90–90-90 targets of the Joint United Nations Program on HIV/AIDS to address Quality of Life (QoL). These targets were originally established to combat the HIV/AIDS pandemic, ensuring that by 2020, 90% of all PLWH would know their serostatus, 90% of those diagnosed would be enrolled in care, and 90% of those in care would attain viral suppression. While significant progress has been made toward achieving these goals, there is a recognition that addressing HIV/AIDS goes beyond mere viral suppression. Hence, the proposal to introduce a fourth goal: where 90% of PLWH have a good health-related QoL. This addition underscores the importance of not only controlling the viral load but also ensuring that the overall well-being and QoL of individuals with HIV are prioritized (8). The World Health Organization defines QoL as an individual’s perspective on life, considering their goals, aspirations, concerns, and expectations (9). Having a good QoL is a key component of healthy aging, however, OPWH are at risk of experiencing poor QoL compared to younger PLWH (10) and compared to older people without HIV (11) due to the presence of multi-morbidity, poly-pharmacy, and the occurrence of geriatric syndromes including frailty, cognitive impairment, falls and reduced physical function (12–15).

The determinants of QoL for OPWH in SSA are complex and differ meaningfully from those in other regions, including social functioning, physical, cognitive, factors related to HIV, caregiving responsibilities, and HIV stigma (16). A recent qualitative study conducted in Uganda revealed that both OPWH and those without HIV shared worries such as the increased risk of non-communicable diseases (NCDs), memory loss, physical pain, decreased energy (17) and the need for social, physical, and financial support to improve their QoL. Studies from the global North report an excess of age-related conditions such as NCDs and geriatric syndromes in OPWH (14, 15, 18, 19). There is limited data from sub-Saharan Africa regarding the impact of geriatric syndromes and other age-related conditions on QoL for OPWH (16). We described the QoL and evaluated the association of socio-demographic, clinical factors, and geriatric syndromes on QoL among OPWH aged 60 years and above, attending the Infectious Disease Institute, Mulago Hospital in Kampala, Uganda.

## Methods

### Study design

We conducted a cross-sectional analysis to assess QoL among the participants of a prospective cohort at enrolment from December 2020 to December 2021. People aged 60 years and above were considered older persons in this study, aligning with the United Nations’ definition which was adopted by Uganda (20).

### Study setting and population

The “Diagnosis and treatment of non-communicable diseases and geriatric syndromes in the HIV Ageing population in sub-Saharan Africa” (HASA cohort) is a prospective observational study of 500 PWH aged ≥60 years. The study participants were recruited from the Adult HIV Clinic of the Infectious Diseases Institute (IDI) in Kampala Uganda, which is a centre of excellence for HIV care and treatment since 2004 (21). IDI clinic attends to complex patients already enrolled or transferred from other public facilities and groups of patients who need particular medical attention, like OPWH in the senior citizens’ clinic. Consecutive patients aged ≥60 who attended the IDI clinic for their routine care were approached and enrolled if willing to participate in the study. At enrolment and during annual follow-up, OPWH are screened within the cohort by a nurse overseen by a physician who also performs routine care.

### Quality of life assessment

Quality of life was assessed using the World Health Organisation Quality of Life OLD instrument (WHOQOL-OLD). WHOQOL-OLD is a multidimensional self-reported questionnaire designed to assess the quality of life in older people aged ≥60 years. It comprises 24 items with a scale range where 24 means the lowest possible or poor QoL to 120 means the highest possible or good QoL. The questions are ranked on a 5-point Likert scale (from not at all, a little, moderate amount, very much to an extreme amount) across 6 domains including; autonomy, social participation, sensory abilities, death and dying, intimacy, and activities of past present future. Items from the sensory abilities and the death and dying facet are reverse-coded items. A maximum score of 20 indicates a good QoL with the domain and a minimum score of 6 indicates a poor QoL. The sensory functions examine how changes in sight, hearing, touch, taste, and appetite affect daily life. Autonomy examines how independence, respect, overall control over life, and the freedom to make your own decisions affect QoL. Past, present and future activities examine past triumphs, old memories, feelings, and future plans. Social participation examines participants’ time use and participation in significant things. Death and dying facet examines how individuals accept death, how inevitable it is, and what it implies. Intimacy, social support and relationships with others were assessed. This tool has been used among older adults living with HIV and without HIV in Uganda (11) and has been validated in similar settings (22).

### Data collection

Study participants provided a detailed medical history, underwent an extensive physical examination and were screened for NCDs and geriatric syndromes. Apart from screening for hypertension and diabetes mellitus, all the data collected in this study is not routinely collected at IDI. The study employed two data management systems, namely Integrated Clinic Enterprise Application (ICEA) and REDCap. ICEA, a module that had been previously used to capture outcomes for a cohort of participants on long-term ART (23). Many fields are mandatory and must be filled in before the record can be considered valid and saved. Moreover, there are internal consistency checks that ensure that the data entered is accurate and falls within the required ranges. Here we collected data on:

- Social demographic characteristics: age, sex, marital status, level of education, and household income.
- HIV related factors: WHO stage, current CD4 count, HIV viral load and duration of ART use.
- Co-medication status which was referred as use of any drugs other than ART.
- Co-morbidities which included hypertension, diabetes, arthritis, cancer and chronic renal disease with glomerular filtration rate of <60 mL/min as per the Chronic Kidney Disease Epidemiology Collaboration (24).

REDCap was used to collect cohort-related data as listed below: a Supplementary Table S1 is also provided with definitions of thresholds for variables assessed in this cohort.

- Depression was assessed using the Patient Health Questionnaire-2 (PHQ-2) and PHQ-9 (25).
- Frailty. The Fried frailty phenotype was used to diagnose frailty, with at least three of these criteria: 1. low physical activity, 2. unintended weight loss, 3. exhaustion, 4. Weakness assessed using hand grip strength and 5. slow walking pace was considered frailty. Pre-frail condition was considered when a participant had one or two of the aforementioned criteria, and robust if one had none (26).
- Nutrition assessment was done using the mini nutrition assessment (MNA), it determines whether the participant is well nourished, at risk of malnutrition or malnourished (27).
- Sarcopenia was assessed using the European Working Group on Sarcopenia in Older People (EWGSOP) and categorized as probable, confirmed and severe sarcopenia. Its evaluation included muscle strength by grip strength, mid arm circumference and gait speed performance (28).
- History of falls were screened using a standard 17-item Questionnaire and we considered any fall in the past 12 months (29).
- Urinary incontinence was assessed based on the frequency, volume, and circumstances of urine leaks.
- The Short Performance Physical Battery (SPPB) was used to assess physical function which included gait speed, a balance test and a chair stand test (30).
- Activities of daily living were screened using the Lawton Instrumental Activities of Daily Living (IADL) Scale to assess daily independence and rated from 0 dependence to 8 autonomy (31).
- The Montreal Cognitive Assessment was used to examine cognitive performance (MoCA). Cognitive impairment was defined as a score < 24 (lowered from 26 to account for cultural differences) (32, 33).
- Disability referred to participants who were; blind, deaf, missing a limb, and/or poliomyelitis.

### Statistical analysis

Mean and median were used to summarise continuous variables related to participant characteristics while frequencies were used to summarise categorical variables. To compare QoL scores, the Mann–Whitney and the Kruskal-Wallis tests were used to compare scores of the individual six domains and the total score of the WHOQOL-OLD stratified by patients’ characteristics. Multiple linear regression models were fitted on log-transformed scores of the WHOQOL-OLD domains and the overall score. We tested for the normality of the residuals by using the Q plot and the Shapiro-Wilcoxson. All the domains and overall QOL fulfilled the normality assumptions of linear regression except the Sensory abilities (SAB) domain, though no special consideration in terms of log transformations was done for SAB so that it could be comparable to the other domains and overall QOL. Associated factors with a *p*-value less than 0.2 were added to the overall and domain QoL models.

A backward elimination was done to select variables significantly associated with different QoL domains. After the backward elimination process emphasis was put on variables that were commonly significantly associated with most of the domains for consistency in presentation of multivariate results of the different domains. A *p*-value of less than 0.05 was be considered statistically significant for all analyses. Data analysis was performed using STATA software (version 16).

## Results

### Social demographic, clinical and geriatric syndromes of the participants

**Figure 1:**
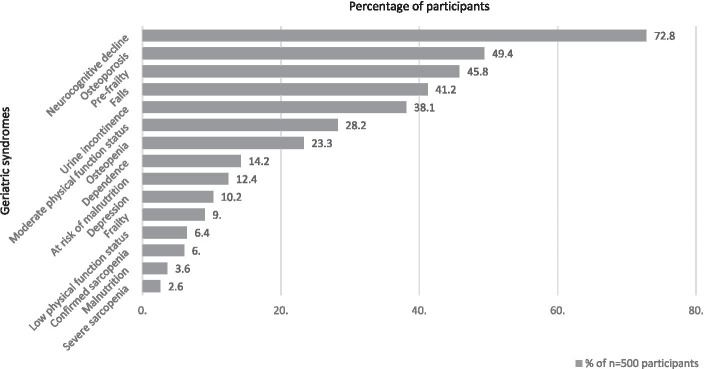
Frequency of geriatric syndromes and age-related conditions among participant.

Five hundred participants were enrolled in the cohort, 51.2% were male and median age was 64 (IQR: 62–68) years. About 34.2% had completed primary level education or higher and 31.2% earned less than the poverty cut-off of 1 dollar per day. Most of our participants (92.2%) had a viral load of <50 copies/ml and the median time on ART was 15 (IQR: 10–17) years. Table 1 further describes the characteristics of the participants. Figure 1 highlights the frequencies of geriatric syndromes and age-related conditions among the 500 participants.

**Table 1:** Socio-demographic and clinical characteristics of older persons with HIV in Kampala.

Participant characteristics	*N* = 500 Mean (SD)	Percentage (%)
**Gender**		
Female	244	48.8
Male	256	51.2
**Age median (IQR)**	**64 (62,68)**	
**Marital status**		
Single/Divorce/Separated/Widowed	270	54.1
Married/In a relationship	229	45.9
**Level of education**		
Completed ordinary level and above	171	34.2
Below ordinary level	329	65.8
**Socio-economic status**		
< 1$ per day	154	31.2
≥ 1$ per day	340	68.8
**WHO stage III and IV**	**345**	**76**
**Current CD4, cells/μL**	**645 (462,850)**	
**Viral load**		
<50copies	459	92.2
<1000copies	496	99.6
**Time on ART, years**	**15 (10,17.0)**	
**Body mass index**		
<18.5	28	5.6
18.5–24.9	254	50.9
>= 25	217	43.5
**Nutrition status**		
At-Risk	62	12.4
Malnourished	18	3.6
**Co-morbidities (HTN, DM, CKD, arthritis)**		
1 NCD	349	69.8
≥ 2 NCDs	151	30.2
**Co-medications**	**2 (0,2)**	
**Number of geriatric syndromes**	**2 (2)**	

### Overall and domain quality of life scores by participant characteristics

Tables 2–4 present comparisons of the WHOQOL-OLD by socio-demographic, clinical and geriatric syndromes among older adult people with HIV at bivariate analysis using correlations and ANOVA tests for significance. WHOQOL-OLD mean score was 90.1 (SD 8.2) out of 120 indicating an overall good QoL. Means (top score of 20) for each domain were: sensory abilities 16.9 (SD 2.7); death and dying 14.9 (SD 3.0); past, present and future activities 15.1 (SD 1.9); autonomy 14.5 (SD 2.1); social participation 14.4 (SD 2.3); intimacy 14.3 (SD 2.2).

**Table 2:** WHOQOL-OLD overall score stratified by sociodemographic characteristics among older people with HIV.

Socio-demographics	Overall score mean (SD)
**Domain score**	90.1 (8.2)
**Gender**	
Female	88.4 (8.5)
Male	91.7 (7.5)
*p*-value	<0.0001
**Age**	−0.056
*p*-value	0.215
**Marital status**	
Single/Divorce/Separated/Widowed	88.6 (8.7)
Married/In a relationship	92.0 (7.2)
*p*-value	<0.0001
**Level of education**	
Primary and above	91.4 (7.9)
Primary level and below	89.4 (8.2)
*p*-value	0.008
**Household income**	
< 1$ per day	87.7 (8.9)
≥ 1$ per day	91.2 (7.6)
*p*-value	<0.0001
**Present tobacco use**	89.6 (6.7)
*p*-value	0.929
**Present alcohol use**	91.2 (7.6)
*p*-value	0.087

SD, standard deviation. WHOQOL-OLD, World Health Organisation quality of life instrument older adults module. SPPB, physical performance battery protocol.

**Table 3:** WHOQOL-OLD overall score stratified by clinic characteristics of older people with HIV.

Clinical characteristics	Overall score mean (SD)
**WHO stage III and IV**	89.8 (8.2)
*p*-value	0.096
**Current CD4, cells/μL**	−0.070
*p*-value	0.121
**Viral load <50 copies**	90.1 (8.1)
*p*-value	0.905
**Time on ART, years**	−0.035
*p*-value	0.440
**Body mass index**	
<18.5	87.0 (9.4)
18.5–24.9	90.8 (8.0)
> = 25	89.8 (8.0)
*p*-value	0.049
**Co-morbidities ¥**	
1 NCD	90.3 (8.5)
≥ 2 NCDs	89.6 (7.6)
*p*-value	0.453
**Co-medications**	0.002
*p*-value	0.966
**Bone density**	
Osteopenia 1 < > 2.5	91.1 (7.8)
Osteoporosis < −2.5	87.5 (9.3)
*p*-value	0.0003
**Disability yes**	85 (11.7)
*p*-value	0.046
**Depressive symptoms**	85.4 (8.4)
*p*-value	<0.0001

SD= standard deviation. WHOQOL-OLD=World Health Organisation quality of life instrument older adults module. ¥ comorbidities: hypertension diabetes mellitus, chronic kidney disease, arthritis.

**Table 4:** WHOQOL-OLD overall score stratified by geriatric syndromes of older people with HIV.

Geriatric syndromes	Overall score mean (SD)
**SPPB/Physical function**	
Low	80.2 (9.3)
Moderate/High	90.2 (7.7)/91.0 (7.7)
*p*-value	<0.0001
**Frailty**	
Pre-frail	89.7 (8.0)
Frail	80.7 (7.5)
*p*-value	<0.0001
**Sarcopenia**	
No	90.5 (7.9)
Probable/Confirmed/Severe	89.6 (6.7)/87.0 (10.5)/85 (11.1)
*p*-value	0.015
**History of falls,** ≥ 1 in the past years	88.8 (8.2)
*p*-value	0.003
**Urine incontinence stress/urge**	87.8 (8.5)
*p*-value	0.007
**Activities of daily living scale (IADL)**	
Dependence	85.0 (9.4)
Autonomy	91.0 (7.7)
*p*-value	<0.0001
**Nutrition status**	
At-Risk	88.4 (8.1)
Malnourished	81.1 (10.3)
*p*-value	<0.0001
**Cognitive impairment <24 points**	89.8 (8.2)
*p*-value	0.247
**Number of geriatric syndromes**	−0.377
*p*-value	<0.0001

SD, standard deviation. WHOQOL-OLD, World Health Organisation quality of life instrument older adults module. SPPB, physical performance battery protocol.

Overall QoL scores were significantly associated with the sex, marital status, level of income, level of education, present alcohol use, depressive symptoms, physical function, frailty, having a disability, sarcopenia, falls, urine incontinence, bone density and activities of daily living. The sensory abilities domain was significantly associated with age, WHO stage, having NCDs, physical function, frailty, sarcopenia, falls, urine incontinence, bone density and daily activities. The Past, Present and Future activities domain was significantly associated with having an income, malnutrition, cognitive impairment, depressive symptoms, physical function, sarcopenia, frailty, bone density, and daily activities. The Death and dying domain was significantly associated with sex, CD4 count, BMI, depressive symptoms, physical function, and history of falls.

### Association of overall and domain quality of life scores by participant characteristics

Table 5 depicts predictors of QoL of OPWH based on participants’ characteristics from the multivariate analysis. Male sex was significantly associated with increased QoL compared to females in the overall QoL score (*p* < 0.01), autonomy (*p* 0.02), death and dying (*p* 0.03) and intimacy (*p* < 0.01) domains. Having an income of ≥1$ per day predicted good QoL compared to those earning ≤1$ per day in the domains of autonomy (*p* 0.03), Past, present, and future activities (*p* 0.03) and intimacy (*p* 0.03). Being malnourished was a determinant of reduced QoL compared to being normal in the overall QoL score (*p* 0.04), and in the domain of social participation (*p* < 0.01). Having ≥2 NCDs predicted good QoL compared to those with 1 or no NCD in the past, present, and future activities domain (*p* 0.01). Having symptoms of depression predicted good QoL compared to those who were not depressed in the domain of sensory abilities (*p* 0.04). Low physical function was a predictor of reduced QoL compared to high physical function in the overall QOL (*p* < 0.01) and most of the QoL domains. Being pre-frail and frail were associated with poor QoL in the overall QoL score (*p* 0.01), Past, present, and future activities (*p* < 0.01), Social participation (*p* < 0.01), death and dying (*p* < 0.01). Frailty further predicted reduced QoL compared to being robust in the overall QOL (*p* < 0.01) and in the domains of sensory abilities (*p* 0.02), autonomy (*p* < 0.01), past, present, and future activities (*p* < 0.01) and social participation (*p* < 0.01).

**Table 5:** Multivariable linear regression models for factors associated with quality of life among older people with HIV.

Patient characteristics	Overall score coefficients (95%CI)
**Gender**	
Female	Ref
Male	2.35 (1.21 – 3.73)**
**Household income**	
< 1$ per day	Ref
≥ 1$ per day	1.30 (−0.16 – 2.76)
**Nutrition status**	
Normal	Ref
At-risk	−0.29 (−2.33 – 1.75)
Malnourished	−2.44 (−6.25 – 1.38)*
**NCDs**	
1 NCD	Ref
≥ 2 NCDs	0.69 (−0.76 – 2.14)
**Depression**	
No depression	Ref
Depression	−1.54 (−3.89 – 0.81)
**SPPB**	
High	Ref
Moderate	0.90 (−0.60 – 2.40)
Low	−4.58 (−7.74 – 1.44)**
**Frailty**	
Robust	Ref
Pre-frail	−1.47 (−2.93 – 0.02)*
Frail	−6.06 (−9.04 – −4.38)**
**Number of geriatric syndromes**	−1.10 (−1.82 – 0.36)**

*significant at 0.05, **significant at 0.01. Coefficients – are from a multivariable linear regression model of quality of life scores adjusting for Gender, Household income, Nutritional status, NCDs, SPPB, and Frailty. 95%CI denotes 95% confidence intervals.

For every unit increase in the number of geriatric syndromes, there was reduced QoL in overall QOL (*p* 0.024), Sensory abilities (*p* < 0.01), and autonomy (*p* 0.01).

## Discussion

This study aimed to evaluate the impact of sociodemographic, clinical features and geriatric syndromes on the QOL for OPWH. Despite high viral suppression (92.2% of participants), all participants in our cohort had at least one NCD and two geriatric syndromes, emphasizing the need for comprehensive geriatric care beyond viral suppression.

In our cohort, we found an overall good QoL among our participants which might have been influenced by the availability of comprehensive HIV programs that offer primary care services. In addition, participants were recruited from a well-established senior citizen clinic (21, 23) integrated within the Infectious Disease Clinic where they receive regular HIV monitoring and management. Similar to our findings, HIV clinics that have integrated geriatric care for OPWH have reported good outcomes for their clients (34). In SSA, there have been reports of good QoL among OPWH with some reporting similar or better QoL of OPWH compared to counterparts living without HIV (35, 36). In contrast, a study done in a community setting in rural Uganda found lower QoL among OPWH compared to those without HIV (11), specifically in the psychosocial domains.

Results depicted that being male and having a daily income of 1 dollar or more per day increased QoL in our population, similar to previous studies (11, 37–39), especially in the ‘autonomy’ and ‘intimacy’ domains. This observation may be attributable to gender differences in cultural and social norms, access to healthcare, jobs and coping strategies in our society. Financial stability may allow access to healthcare, nutritious food, social relations and other factors that contribute to the well-being of this population.

While the presence of multiple NCDs is generally burdensome for PLWH and negatively impact their QoL and mortality risk (12), our study found that having >2 NCDs increased QoL in the ‘past, present, and future activities’ domain. This odd result suggests a potential influence of specialized clinic care (20), frequent visits, and proactive healthcare-seeking behavior among OPWH with co-morbidities in our setting, warranting further exploration.

Notably, depressive symptoms decreased QoL in our study, specifically the domains of intimacy, autonomy, and overall QoL. These findings align with previous studies that have demonstrated the negative impact of depression (10, 38) on QoL among older individuals.

This is the first study to evaluate how geriatric syndromes impact the QoL of OPWH in a SSA context. Neurocognitive decline, pre-frailty, falls, urine incontinence, and low physical function status were the most common geriatric syndromes in our cohort. However, the finding of a high degree of cognitive impairment in this study needs to be further investigated.

As anticipated, our findings revealed that pre-frailty, frailty, malnourishment, depressive symptoms, and low to moderate physical function each independently decreased multiple domains and the overall QoL for OPWH. Specifically, both frailty and low physical function were associated with a decrease in at least three domains and the overall QoL for OPWH. These factors are interrelated, often co-occur and may manifest bi-directional influences. We found that with each additional geriatric syndrome, there is a corresponding decrease in QoL. Specifically, this decrease was observed in overall, sensory abilities and autonomy QoL.

For instance, malnutrition can substantially contribute to the emergence and progression of frailty and reduced physical function, especially among older adults (40). Reduced physical function in OPWH may lead to reduced mobility and physical activity, limiting their ability to engage in daily tasks and activities. This can result in a decreased sense of independence and an overall decrease in their QoL. Despite a small percentage of participants being at risk or experiencing malnutrition, our study identified its impact on the social participation domain and the overall QoL of OPWH. Participants with low functional status had decreased QoL in multiple domains including; autonomy, social participation, death and dying, past present and future activities, and the overall QoL. Low physical function increases the risk of falls and injuries, further hampering their physical capabilities and potentially leading to long-term disabilities.

### Limitations

Our study limitation is the lack of a comparison group of people without HIV therefore we cannot directly attribute levels of QoL to HIV infection, but they could be common findings in older age (36). Most of our participants had a primary level or higher education, were on antiretroviral therapy for about 15 years and were recruited from a specialized HIV care facility with a focus on older adults, our results might not fully represent the broader Ugandan population. But may represent populations with integrated geriatric care (34).

The strength of our study is the use of a cohort with a large number of OPWH with well-balanced gender representation.

## Conclusion

As PLWH live longer, there is a need to incorporate the screening and management of geriatric syndromes into routine HIV care, given their impact on QoL. Assessing geriatric syndromes can facilitate timely interventions like dietary adjustments, nutritional supplementation, and targeted exercise programs, aiming to enhance QoL. It is important to recognize that factors decreasing QoL in this context may differ from other settings, underscoring the importance of evaluating QoL according to the local socio-economic and healthcare frameworks. Furthermore, determining the feasibility of integrating geriatric care, including considerations like task shifting or consultations, into HIV clinics may be considered in resource-limited settings. Our findings also underscore the importance of demographic variables when considering improving QoL outcomes. Addressing disparities based on gender and income levels can be important in the overall well-being of OPWH.

## Data availability statement

The raw data supporting the conclusions of this article will be made available by the authors, without undue reservation.

## Ethics statement

The studies involving humans were approved by Joint Clinic and Research Centre Committee Center (JC 1319) and the Uganda National Council for Science and Technology (HS454ES). The studies were conducted in accordance with the local legislation and institutional requirements. The participants provided their written informed consent to participate in this study.

## Author contributions

ES: Methodology, Writing – original draft, Writing – review & editing. PM: Data curation, Methodology, Writing – review & editing. GB: Formal analysis, Methodology, Writing – review & editing. SN: Data curation, Investigation, Writing – review & editing. BC: Conceptualization, Funding acquisition, Investigation, Project administration, Resources, Supervision, Writing – review & editing.
