# Supplementary material for: The impact of geriatric syndromes on quality of life among older people living with HIV in Kampala, Uganda
Source: Front Public Health. 2024 Jan 23;12:1306151. doi: 10.3389/fpubh.2024.1306151 (PMC10845335; doi:10.3389/fpubh.2024.1306151)
Supplement: Supplementary file 1 [file Table_1.DOCX]

**Supplementary tables for cohort evaluations**

**Table 1.** Evaluations of non-communicable diseases in the cohort participants.

| **Evaluation** | **Details** | **Interpretation** |
| --- | --- | --- |
| Medication regimen monitoring | Pharmacy prescriptions for ART and pharmacy prescription or self- report for all other co-medications including herbal medications. | ≥5 drugs other than ART interpreted as polypharmacy |
| Renal disease screening | Estimated Glomerular Filtration Rate (GFR) using the Chronic Kidney Disease (CKD) Epidemiology Collaboration 2009 Equation | Impaired renal function: GFR <60 mL/minute |
| Hypertension | Considered if on anti-hypertensive medication or average blood pressure of two readings ≥140/90 mmHg. If the average was ≥140/90, a third reading was taken for confirmation(43) |  |
| Diabetes Mellitus | Considered if on antidiabetic medication and if elevated blood glucose (non-fasting ≥ 11.1 mmol/L or fasting ≥ 7 mmol/L) in the presence of symptoms. If elevated values in someone asymptomatic; a repeat fasting blood sugar was performed on a subsequent day to confirm the diagnosis. |  |
| Arthritis | Self-report of current symptoms or treatment for arthritis and validated with medical records when available |  |
| Cancer | Non-HIV related cancers diagnosed by participants’ self -report and validated with medical records |  |
| Bone density screening | Bone density screening using the Calcaneal Quantitative Ultrasound (cQUS). | Osteopenia:  T score: -1 and -2.5  Osteoporosis:  T score <-2.5 |

ART, anti-retroviral therapy; WHO, World Health Organization; ISH, International Society for Hypertension; COPD, Chronic Obstructive Pulmonary Disease; PEFR, Peak expiratory flow rate; GFR, Glomerular filtration rate; CKD, chronic kidney disease; cQUS, calcaneal quantitative ultra sound;

Table 2. Evaluations of geriatric syndromes in the cohort participants

**Table 2.** Evaluations of geriatric syndromes in the cohort participants.

| **Evaluation** | **Details** | **Interpretation** |
| --- | --- | --- |
| Physical function | The Short Performance Physical Battery (SPPB) measuring balance, gait speed and chair stands. The three components are scored for 0-4 using standardized and validated criteria. | Low physical performance: 0-6, moderate physical performance: 7-9, high physical performance: 10-12 |
| Sarcopenia | Assessed using criteria by the European Working Group on Sarcopenia in Older People (EWGSOP2)  1) Muscle strength by grip strength with handheld dynamometry (<27kg for men, <16Kg for women)  2) Mid arm muscle circumference by skinfold thickness and limb circumferences ∗∗, cut off: <21.1 cm men and <19.2 cm women or calf circumference <31 cm  3) Gait speed performance by SPPB (cut off: < 0.8 m/sec). | If  1: probable sarcopenia  If 1 and 2: confirmed sarcopenia  If 1, 2 and 3: severe sarcopenia |
| Frailty | Scoring proposed by Fried and colleagues.  1)Weight loss (unintentional ≥4.5kg or documented >5% in 12 months)  2) Grip strength: cut off lowest 20% adjusted for gender and BMI  3) Self-reported exhaustion using two items from the Centre for Epidemiological Studies Depression Scale (CES-D) scale with an answer of moderate amount of the time (3–4 days) or/and most or all of the time (5–7 days). 4) Physical activity: reporting “Limited a lot” when asked whether their health limits vigorous activities  5) Slowness: time in seconds to complete a 4-m walk by gender and height specific cut offs | Frail: ≥3 criteria; pre-frail: 1 or 2 criteria; non-frail: 0 criteria present |
| Cognitive impairment | The Montreal Cognitive Assessment (MoCA) [53]  https://www.mocatest.org/ | Cognitive impairment defined as a score <24 (lowered from 26 to account for cultural differences (31)) |
| Falls history | Standard 17 items Questionnaire for Falls history . Includes activities prior falling, perceived causes, environmental factors and injuries sustained. | Fallers: ≥ one fall in the past 2 years with the most serious fall considered for analysis |

∗∗ Mid arm muscle circumference: Mid arm circumference (cm)- (3.142 x triceps skin fold thickness(mm))

SPPB, Short physical performance battery; CES-D, Centre for Epidemiological Studies Depression Scale; MoCA, Montreal cognitive assessment; PHQ, Patient health questionnaires; IADL, Instrumental activities of daily living.

Supplementary for **table 2.** WHOQOL-OLD domain scores stratified by sociodemographic characteristics among OPWH

| **SOCIO-DEMOGRAPHICS** | **DOMAINS OF WHOQOL-OLD** | | | | | |
| --- | --- | --- | --- | --- | --- | --- |
|  | **SAB** | **AUT** | **PPF** | **SOP** | **DAD** | **INT** |
|  | **Mean (SD)** | **Mean (SD)** | **Mean (SD)** | **Mean (SD)** | **Mean (SD)** | **Mean (SD)** |
| **Domain Score** | 16.9(2.7) | 14.5(2.1) | 15.1(1.9) | 14.4(2.3) | 14.9(3.0) | 14.3(2.2) |
| **Gender** |  |  |  |  |  |  |
| Female | 16.8(2.8) | 14.1(2.3) | 15.0(1.8) | 14.3(2.4) | 14.6(3.2) | 13.6(2.2) |
| Male | 17.0(2.6) | 14.8(1.9) | 15.2(1.8) | 14.6(2.2) | 15.2(2.8) | 14.9(1.9) |
| **P-value** | 0.287 | 0.0001 | 0.328 | 0.060 | 0.036 | <0.0001 |
| **Age** | -0.104 | -0.157 | 0.005 | 0.043 | 0.046 | -0.020 |
| **P-value** | 0.020 | 0.0004 | 0.911 | 0.336 | 0.304 | 0.664 |
| **Marital status** |  |  |  |  |  |  |
| Single/Divorce/Separated/Widowed | 16.8(2.8) | 14.2(2.3) | 15.0(1.8) | 14.2(2.4) | 14.8(3.1) | 13.5(2.2) |
| Married/ In a relationship | 17.1(2.6) | 14.8(1.9) | 15.2(1.8) | 14.8(2.1) | 15.0(2.9) | 15.2(1.7) |
| **P-value** | 0.262 | 0.002 | 0.439 | 0.018 | 0.282 | <0.0001 |
| **Level of Education** |  |  |  |  |  |  |
| Primary and above | 17.1(2.7) | 14.9(2.0) | 15.2(1.8) | 14.5(2.4) | 15.1(3.0) | 14.6(2.1) |
| Primary level and below | 16.8(2.8) | 14.2(2.1) | 15.0(1.8) | 14.4(2.2) | 14.8(3.1) | 14.1(2.2) |
| **P-value** | 0.187 | 0.001 | 0.278 | 0.623 | 0.321 | 0.030 |
| **Household income** |  |  |  |  |  |  |
| < 1$ per day | 16.7(2.8) | 13.9(2.1) | 14.7(1.8) | 13.9(2.3) | 14.7(3.0) | 13.7(2.2) |
| ≥ 1$ per day | 17.0(2.7) | 14.7(2.1) | 15.3(1.8) | 14.7(2.3) | 15.0(3.0) | 14.6(2.1) |
| **P-value** | 0.268 | <0.0001 | 0.0014 | 0.001 | 0.408 | <0.0001 |
| **Present Tobacco use** | 18.3(1.4) | 14.1(2.8) | 16(1.2) | 14(2) | 13.7(3.5) | 13.4(1.7) |
| **P-value** | 0.393 | 0.227 | 0.298 | 0.714 | 0.570 | 0.279 |
| **Present Alcohol use** | 17.2(2.5) | 14.9(2.1) | 15.1(1.7) | 14.6(2.2) | 15.3(2.6) | 14.4(2.1) |
| **P-value** | 0.165 | 0.703 | 0.921 | 0.073 | 0.185 | 0.566 |

SD= Standard Deviation. WHOQOL-OLD=World Health Organisation Quality of Life Instrument Older Adults Module. SPPB =Physical performance Battery Protocol. SAB=sensory abilities, AUT=autonomy, PPF= past, present, and future activities, SOP=social participation, DAD=death and dying, and INT=intimacy

Supplementary for t**able 3.** WHOQOL-OLD domain scores stratified by clinic characteristics of OPWH

| **CLINICAL CHARACTERISTICS** | **DOMAINS OF WHOQOL-OLD** | | | | | |
| --- | --- | --- | --- | --- | --- | --- |
|  | **SAB** | **AUT** | **PPF** | **SOP** | **DAD** | **INT** |
|  | **Mean (SD)** | **Mean (SD)** | **Mean (SD)** | **Mean (SD)** | **Mean (SD)** | **Mean (SD)** |
| **WHO Stage III and IV** | 16.9(2.8) | 14.4(2.0) | 15.1(1.8) | 14.4(2.3) | 14.9(2.9) | 14.3(2.2) |
| **P-value** | 0.045 | 0.273 | 0.522 | 0.306 | 0.674 | 0.440 |
| **Current CD4,**  **cells/µL** | -0.011 | -0.039 | -0.018 | -0.006 | -0.101 | -0.020 |
| **P-value** | 0.814 | 0.387 | 0.683 | 0.892 | 0.025 | 0.664 |
| **Viral load <50copies** | 16.9(2.7) | 14.5(2.1) | 15.1(1.8) | 14.4(2.3) | 14.9(3.0) | 14.2(2.2) |
| **P-value** | 0.397 | 0.468 | 0.342 | 0.697 | 0.351 | 0.161 |
| **Time on ART, years** | -0.047 | -0.026 | 0.031 | -0.025 | -0.034 | -0.012 |
| **P-value** | 0.291 | 0.570 | 0.497 | 0.584 | 0.451 | 0.798 |
| **Body Mass Index** |  |  |  |  |  |  |
| <18.5 | 17.1(2.6) | 13.7(1.9) | 14.8(1.7) | 13.8(2.8) | 13.8(3.3) | 13.9(2.1) |
| 18.5-24.9 | 16.9(2.8) | 14.6(2.1) | 15.1(1.8) | 14.5(2.1) | 15.2(2.8) | 14.5(2.1) |
| >= 25 | 16.9(2.7) | 14.4(2.1) | 15.1(1.9) | 14.5(2.4) | 14.7(3.1) | 14.2(2.2) |
| **P-value** | 0.952 | 0.068 | 0.674 | 0.317 | 0.016 | 0.105 |
| **Co-morbidities ¥** |  |  |  |  |  |  |
| 1 NCD | 17.1(2.6) | 14.6(2.1) | 15.0(1.8) | 14.4(2.3) | 14.9(3.1) | 14.4(2.2) |
| ≥ 2 NCDs | 16.5(2.9) | 14.2(2.1) | 15.3(1.8) | 14.6(2.4) | 14.9(2.9) | 14.1(1.9) |
| **P-value** | 0.041 | 0.071 | 0.096 | 0.319 | 0.956 | 0.190 |
| **Co-medications** | -0.027 | -0.034 | 0.072 | 0.036 | -0.034 | 0.001 |
| **P-value** | 0.554 | 0.449 | 0.110 | 0.427 | 0.450 | 0.977 |
| **Bone Density** |  |  |  |  |  |  |
| Osteopenia 1<>2.5 | 17.0(2.6) | 14.6(2.1) | 15.4(1.7) | 14.7(2.1) | 14.9(2.9) | 14.5(2.0) |
| Osteoporosis <-2.5 | 16.3(3.1) | 14.0(2.3) | 14.7(2.1) | 13.9(2.6) | 14.9(2.9) | 13.7(2.2) |
| **P-value** | 0.020 | 0.048 | 0.007 | 0.012 | 0.709 | 0.002 |
| **Disability (**yes) | 15.7(4.3) | 14.1(1.8) | 14(2.2) | 12.2(3.4) | 14.9(4.1) | 14.1(3.1) |
| **P-value** | 0.161 | 0.582 | 0.051 | 0.002 | 0.997 | 0.792 |
| **Depressive symptoms** | 16.5(2.7) | 13.4(2.2) | 14.4(2.2) | 13.5(2.8) | 14.2(3.1) | 13.4(2.4) |
| **P-value** | 0.329 | 0.0001 | 0.005 | 0.003 | 0.065 | 0.002 |

SD= Standard Deviation, SAB=sensory abilities, AUT=autonomy, PPF= past, present, and future activities, SOP=social participation, DAD=death and dying, and INT=intimacy. WHOQOL-OLD=World Health Organisation Quality of Life Instrument Older Adults Module ¥ comorbidities: hypertension diabetes mellitus, chronic Kidney disease, arthritis

Supplementary for **table 4.** WHOQOL-OLD domain scores stratified by geriatric syndromes of OPWH

| **GERIATRIC SYNDROMES** | **DOMAINS OF WHOQOL-OLD** | | | | | |
| --- | --- | --- | --- | --- | --- | --- |
|  | **SAB** | **AUT** | **PPF** | **SOP** | **DAD** | **INT** |
|  | **Mean (SD)** | **Mean (SD)** | **Mean (SD)** | **Mean (SD)** | **Mean (SD)** | **Mean (SD)** |
| **SPPB** |  |  |  |  |  |  |
| Low | 15.2(3.5) | 12.6(2.9) | 13.6(2.4) | 13.4(3.0) | 12.9(3.5) | 13.4(2.5) |
| Moderate | 17.0(2.6) | 14.4(1.9) | 15.1(1.8) | 14.4(2.4) | 15.2(2.9) | 14.1(2.1) |
| High | 17.1(3.5) | 14.7(2.0) | 15.3(1.7) | 14.7(2.1) | 14.9(3.0) | 14.4(2.1) |
| **P-value** | 0.001 | <0.0001 | <0.0001 | <0.0001 | 0.001 | 0.020 |
| **Frailty** |  |  |  |  |  |  |
| Pre-frail | 16.8(2.6)) | 14.3(2.1) | 15.1(1.8) | 14.5(2.1) | 13.9(2.8) | 14.1(2.2) |
| Frail | 15.2(3.5) | 12.5(2.2) | 13.6(2.2) | 11.9(2.8) | 15.0(3.0) | 13.3(2.3) |
| **P-value** | <0.0001 | <0.0001 | <0.0001 | <0.0001 | 0.079 | 0.0001 |
| **Sarcopenia** |  |  |  |  |  |  |
| No | 16.9(2.6) | 14.5(2.0) | 15.2(1.8) | 14.6(2.2) | 14.9(3.1) | 14.3(2.1) |
| Probable | 17.3(2.5) | 13.7(2.8) | 13.4(1.2) | 14.3(2) | 15.6(2.4) | 15.2(1.9) |
| Confirmed | 16.3(3.4) | 13.8(2.6) | 14.6(1.7) | 13.4(2.9) | 14.7(2.8) | 14.1(2.5) |
| Severe | 15.1(3.7) | 13.8(3.0) | 14.3(1.8) | 13.2(2.9) | 14.7(2.7) | 14(1.9) |
| **P-value** | 0.056 | 0.098 | 0.004 | 0.010 | 0.883 | 0.557 |
| **History of falls** | 16.5(2.7) | 14.2(2.1) | 15.1(1.7) | 14.3(2.4) | 14.6(3.0) | 14.1(2.1) |
| **P-value** | 0.003 | 0.026 | 0.730 | 0.412 | 0.052 | 0.082 |
| **Urine incontinence** | 16.2(2.8) | 14.1(2.3) | 15.0(2.0) | 14.1(2.5) | 14.5(2.9) | 13.9(2.3) |
| **P-value** | 0.007 | 0.262 | 0.346 | 0.596 | 0.266 | 0.006 |
| **IADL** |  |  |  |  |  |  |
| Dependence | 15.3(3.5) | 13.1(2.4) | 14.7(2.0) | 13.3(2.6) | 14.8(3.0) | 13.8(2.2) |
| Autonomy | 17.2(2.5) | 14.7(2.0) | 15.2(1.8) | 14.6(2.2) | 14.9(3.0) | 14.4(2.1) |
| **P-value** | <0.0001 | <0.0001 | 0.025 | <0.0001 | 0.684 | 0.059 |
| **Nutrition status** |  |  |  |  |  |  |
| At-Risk | 16.6(3.0) | 14.0(2.1) | 15.0(1.8) | 14.1(2.4) | 14.9(3.0) | 13.9(2.2) |
| Malnourished | 15.7(3.1) | 12.6(2.3) | 13.9(1.7) | 11.6(2.3) | 13.6(3.6) | 13.6(2.9) |
| **P-value** | 0.102 | 0.0001 | 0.014 | <0.0001 | 0.187 | 0.105 |
| **Cognitive impairment** | 16.7(2.8) | 14.3(2.1) | 15.1(1.7) | 14.6(2.2) | 14.9(3.1) | 141(2.1) |
| **P-value** | 0.091 | 0.003 | 0.600 | 0.046 | 0.818 | 0.022 |
| **Number of geriatric syndromes** | -0.283 | -0.338 | -0.165 | -0.215 | -0.134 | -0.195 |
| P-value | <0.0001 | <0.0001 | 0.0002 | <0.0001 | 0.003 | <0.0001 |

SD= Standard Deviation, SAB=sensory abilities, AUT=autonomy, PPF= past, present, and future activities, SOP=social participation, DAD=death and dying, and INT=intimacy. WHOQOL-OLD=World Health Organisation Quality of Life Instrument Older Adults Module. SPPB =Physical performance Battery Protocol. Activities of daily living scale (IADL)

Supplementary for t**able 5.** Multivariable linear regression models for factors associated with quality of life (expressed as individual domains) among OPWH

| **PATIENT CHARACTERISTICS** | **Model 1:**  **SAB** | **Model 2**  **AUT** | **Model 3**  **PPF** | **Model 4**  **SOP** | **Model 5**  **DAD** | **Model 6**  **INT** |
| --- | --- | --- | --- | --- | --- | --- |
|  | **Coefficients (95%CI)** | **Coefficients (95%CI)** | **Coefficients (95%CI)** | **Coefficients (95%CI)** | **Coefficients (95%CI)** | **Coefficients (95%CI)** |
| **Gender** |  |  |  |  |  |  |
| Female | Ref | Ref | Ref | Ref | Ref | Ref |
| Male | -0.04  (-0.53 — 0.46) | 0.41  (0.04 — 0.78)* | 0.01  (-0.31 — 0.34) | 0.21  (-0.20 — 62) | 0.63  (0.05 — 1.20)* | 1.08  (0.69 —1.46)** |
| **Household income** |  |  |  |  |  |  |
| < 1$ per day | Ref | Ref | Ref | Ref | Ref | Ref |
| ≥ 1$ per day | -0.06  (-0.59 — 0.47) | 0.43  (0.04 — 0.83)* | 0.38  (0.04 — 0.73)* | 0.35  (-0.08 — 0.78) | -0.17  (-0.77 — 0.44) | 0.45  (0.04 — 0.86)* |
| **Nutrition status** |  |  |  |  |  |  |
| Normal | Ref | Ref | Ref | Ref | Ref | Ref |
| At-risk | -0.001  (-0.73 — 0.73) | -1.15  (-0.70 — 0.40) | 0.17  (-0.32 — 0.65) | -0.08  (-0.69 — 0.52) | 0.09  (-0.75 — 0.93) | -0.31  (-0.88 — 0.26) |
| Malnourished | 0.58  (-0.80-1.95) | -0.31  (-1.34 — 7.13) | -0.25  (-1.16 — 0.65) | -1.65  (-2.78 — 0.52)** | -0.70  (-2.27 — 0.88) | -0.03  (-1.09 — 1.04) |
| **NCDs** |  |  |  |  |  |  |
| 1 NCD | Ref | Ref | Ref | Ref | Ref | Ref |
| ≥ 2 NCDs | -0.33(-0.85 — 0.19) | -0.11  (-0.50 — 0.28) | 0.45  (0.10 — 0.79)* | 0.36  (-0.07 — 0.76) | 0.31  (-0.29 — 0.90) | 0.03  (-0.37 — 0.43) |
| **Depression** |  |  |  |  |  |  |
| No depression | Ref | Ref | Ref | Ref | Ref | Ref |
| Depression | 1.01  (0.16 — 1.85)* | -0.28  (-0.91 — 0.35) | -0.57  (-1.13 — 0.01)* | -0.46  (-1.16 — 0.24) | -0.59  (-1.56 — 0.38) | -0.60  (-1.26 — -0.06) |
| **SPPB** |  |  |  |  |  |  |
| High | Ref | Ref | Ref | Ref | Ref | Ref |
| Moderate | 0.47  (-0.07 — 1.01) | 0.06  (-0.34 — 0.47) | 0.08  (-0.28 — 0.44) | 0.04  (-0.40 — 0.49) | 0.31  (-0.31 — 0.93) | -0.05  (-0.47 — 0.37) |
| Low | 0.46  (-0.67 — 1.59) | -0.43  (-1.27 — 0.42) | -1.48  (-2.18 — -0.77)* | -1.41  (-2.35 — -0.48)** | -1.65  (-2.95 — -0.36)* | -0.16  (-1.04 — 0.72) |
| **Frailty** |  |  |  |  |  |  |
| Robust | Ref | Ref | Ref | Ref | Ref | Ref |
| Pre-frail | -0.8  (-0.91 — 0.14) | -0.32  (-0.71 — 0.07) | -1.48  (-69 — 001)* | -0.31  (-0.74 — 0.12) | 0.24  (-0.36 — 0.84) | -0.32  (-0.73 — -0.09) |
| Frail | -1.17  (-2.25 —0.10)* | -1.04  (-1.84 — -0.24)* | -0.358  (-0.28 — -0.61)** | -2.15  (-3.04 — -1.26)** | 0.19  (-1.04 — 1.42) | -0.37  (-1.20 — 0.47) |
| **Number of geriatric syndromes** | -0.73(-0.10— 0.47)* | -0.38(-0.58— 0.18)** | 0.15(-0.02 — 0.33) | 0.11(-0.10 — 0.33) | -0.15(-0.45 — 0.16) | -0.12  (0.33 — 0.08) |

*significant at 0.05 **significant at 0.01

SAB = sensory abilities, AUT = autonomy, PPF past, present, and future activities, SOP = social participation, DAD = death and dying, and INT = intimacy. WHOQOL OLD = World Health Organization Quality of Life Instrument Older Adults Module.

Model 1 represents a multivariate linear regression model for quality of life (WHOQOL-OLD) scores for sensory abilities (SAB).

Model 2 represents a multivariate linear regression model for quality of life (WHOQOL-OLD) scores for autonomy (AUT).

Model 3 represents a multivariate linear regression model for quality of life (WHOQOL-OLD) scores for past, present, and future activities (PPF).

Model 4 represents a multivariate linear regression model for quality of life (WHOQOL-OLD) scores for social participation (SOP).

Model 5 represents a multivariate linear regression model for quality of life (WHOQOL-OLD) scores for death and dying (DAD).

Model 6 represents a multivariate linear regression model for quality of life (WHOQOL-OLD) scores for intimacy (INT).

Coefficients – are from a multivariable linear regression model of quality of life scores adjusting for Gender, Household income, Nutritional status, NCDs, SPPB, and Frailty.

95%CI denotes 95% confidence intervals.
